# Pharmacotherapy for hypertension in Sub-Saharan Africa: a systematic review and network meta-analysis

**DOI:** 10.1186/s12916-020-01530-z

**Published:** 2020-03-27

**Authors:** Anna Seeley, Josephine Prynn, Rachel Perera, Rebecca Street, Daniel Davis, Anthony O. Etyang

**Affiliations:** 1 Medical Research Council Unit Lifelong Health and Ageing at UCL, Department of Population Science and Experimental Medicine, University College London, London, UK; 2 Nuffiend Department of Primary Health Care Sciences, Woodstock Road, Oxford, OX2 6GG UK; 3 University College London, London, UK; 4 Department of Epidemiology and Demography, KEMRI Wellcome Trust Research Programme, Kilifi, Kenya

**Keywords:** Hypertension, Raised blood pressure, Sub-Saharan Africa, Africa, Antihypertensive agents, Hypertension/therapy, Combination therapy

## Abstract

**Background:**

The highest burden of hypertension is found in Sub-Saharan Africa (SSA) with a threefold greater mortality from stroke and other associated diseases. Ethnicity is known to influence the response to antihypertensives, especially in black populations living in North America and Europe. We sought to outline the impact of all commonly used pharmacological agents on both blood pressure reduction and cardiovascular morbidity and mortality in SSA.

**Methods:**

We used similar criteria to previous large meta-analyses of blood pressure agents but restricted results to populations in SSA. Quality of evidence was assessed using a risk of bias tool. Network meta-analysis with random effects was used to compare the effects across interventions and meta-regression to explore participant heterogeneity.

**Results:**

Thirty-two studies of 2860 participants were identified. Most were small studies from single, urban centres. Compared with placebo, any pharmacotherapy lowered SBP/DBP by 8.51/8.04 mmHg, and calcium channel blockers (CCBs) were the most efficacious first-line agent with 18.46/11.6 mmHg reduction. Fewer studies assessing combination therapy were available, but there was a trend towards superiority for CCBs plus ACE inhibitors or diuretics compared to other combinations. No studies examined the effect of antihypertensive therapy on morbidity or mortality outcomes.

**Conclusion:**

Evidence broadly supports current guidelines and provides a clear rationale for promoting CCBs as first-line agents and early initiation of combination therapy. However, there is a clear requirement for more evidence to provide a nuanced understanding of stroke and other cardiovascular disease prevention amongst diverse populations on the continent.

**Trial registration:**

PROSPERO, CRD42019122490. This review was registered in January 2019.

## Background

Hypertension is the leading global risk factor for death, accounting for 13% of mortality [1]. Recent epidemiological trends show that the burden of disease in Sub-Saharan Africa (SSA) has overtaken many European and North American states [2]. In large urban areas, 30–50% of adults are classed as hypertensive [3]; prevalence in rural areas is 15–25% [4–6]. Hypertension is the most important modifiable risk factor for stroke, which accounts for up to 11% of adult deaths in Sub-Saharan Africa [7]. Management of both the acute and chronic consequences of hypertension remains poorly optimised across much of the continent, with delays in the presentation, limited access to diagnostic imaging services and rudimentary follow-up care [8–10].

Only 25% of countries have a national framework for hypertension, and just 7% of those with hypertension in Africa achieve control [11]. The Pan-African Society for Cardiovascular Disease guidelines provide a roadmap towards the WHO non-communicable disease target of 25% reduction in high blood pressure by 2025. Though pragmatic, these are predominantly based upon European and American guidelines, with little reference to research conducted on the continent. It is well established that black people living in the Northern Hemisphere respond differently to antihypertensive agents compared to white populations due to a variety of phenotypic differences including lower circulating renin and higher concentrations of skeletal muscle creatine kinase [12]. Studies in African-Americans are not necessarily generalisable to SSA populations, due to both genetic diversity and contrasting environmental settings. Emerging evidence suggests that hypertension in SSA is more severe, more resistant to treatment and more likely to lead to premature morbidity and mortality [13]. Additionally, many African states are resource-limited and focus on public health strategies on maximising distribution of a restricted formulary, to minimise costs and improve consistency of services [14–16]. Delivery of care is common by lower-skilled healthcare workers, especially in rural localities, and access to medical care may require high out-of-pocket expenditure [17].

There have been no systematic reviews to comprehensively synthesise the efficacy of pharmacotherapy in the treatment of hypertension in SSA. African populations are also underrepresented in global trials and meta-analysis. Two previous reviews do make some specific reference to the continent; however, comparisons are limited to monotherapy only [18, 19]. In light of this, we aimed to review the evidence for all common pharmacological treatments, for persons living in SSA, with regard to both the reduction in blood pressure and prevention of associated cardiovascular disease.

## Methods

### Search strategy and selection criteria

We used the Preferred Reporting Items for Systematic Reviews and Meta-Analyses (PRISMA) guidelines, and the review was prospectively registered on PROSPERO (CRD42019122490). Full details of our search strategy can be found in Additional file 1. We conducted a search on MEDLINE, EMBASE, Cochrane Reviews and the African Index Medicus in January 2019 with the terms ‘antihypertensive agents’ or ‘hypertension’ or any of the antihypertension drug classes or individual drug names, as listed in the British National Formulary (BNF). This was combined with ‘Sub-Saharan Africa’ or any of the regions or individual countries as listed on the United Nations. We restricted our results to randomised control trials, clinical control trials or clinical trials. We did not place any language or date restrictions. Relevant meta-analyses were hand-searched for any other relevant trials. References were imported and managed on Covidence (www.covidence.org, Veritas Health Innovation Ltd.).

As per previous reviews [18], trials over a 2-week duration were eligible for inclusion. We did not place any limits on study size. International multi-centre trials were considered as long as there were individual data available for SSA and the population studied in SSA was representative of the population’s racial distribution. For example, trials conducted in South Africa were excluded where < 10% of the participants were black or of mixed race. We only included trials with agents approved by the Food and Drug Association, and in current production, or listed in the British National Formulary [20] or WHO Essential Medicines List [21].

### Data extraction and quality assessment

Covidence was used to manage the abstract and full-text screening. Three researchers (AS, RP, RS) screened all abstracts and then full texts in duplicate to extract those meeting our inclusion criteria. Where conflict arose, papers were discussed with the senior author (AE). Three authors (AE, AS, JP) designed a standardised data extraction template, to include characterisation about study population and geography; change in blood pressure in each intervention arm; reduction in all-cause mortality or cardiovascular mortality and morbidity; and reported adverse events. Data extraction was performed by one reviewer (AS), but extracted data was cross-checked against raw data on a second occasion. All outlying results were reviewed a third time for possible translation errors. Where data were not available, we contacted the study authors and allow an 8-week period for response.

For quality assessment, we used the revised Cochrane ‘Risk of Bias 2’ tool [22]. Each study was independently assessed by at least two reviewers (AS, JP, RP, RS) using standardised decision trees and data input sheets. Studies were ranked as ‘low’, ‘some concerns’ or ‘high’ risk of bias with regard to selection bias (randomisation and allocation concealment), performance bias (blinding of participants and investigators), detection bias (blinding of outcome adjudicators), attrition bias (differential loss to follow-up) and reporting bias (selective outcome reporting) and then given an overall judgement. Where there was disagreement, this was discussed as a panel.

### Statistical analysis

Statistical analysis was completed in STATA 15 (StataCorp, TX). We extracted the mean change in blood pressure in each treatment arm with standard deviation (SD). Where this was not available, we calculated this from baseline and end blood pressures and corresponding SDs, using unpaired two-tailed *t* tests. If there were no measures of variance quoted, we estimated these using a multiple imputation model based on the sample size and average standard deviation observed across all trials and pooling estimates (minimum imputations = 10).

We performed a random effects meta-analysis because the age span and geographical distribution of our studies were wide. We divided our studies into those comparing monotherapy with a placebo, a different monotherapy regime and a combination therapy. We presented a change in systolic (SBP) and diastolic (DBP) blood pressures by intervention and grouped it by class. For ACE inhibitor (ACEi), beta-blocker (BB), calcium channel blocker (CCB), diuretic monotherapy and placebo regimes, we then performed a network meta-analysis to assess the relative efficacy and ranking across classes. We again used a random effects model, explored global and nodal sources of inconsistency and presented our results in terms of overall network geometry and ranking of treatments. We performed meta-regression analysis to explore the potential effects of different demographics, publication date, study quality and study design on our results. A funnel plot was used to further examine the publication bias. Finally, we performed a sensitivity analysis to explore how either imputed results or risk of bias may have affected our conclusions.

## Results

### General characteristics of studies

**Fig. 1:**
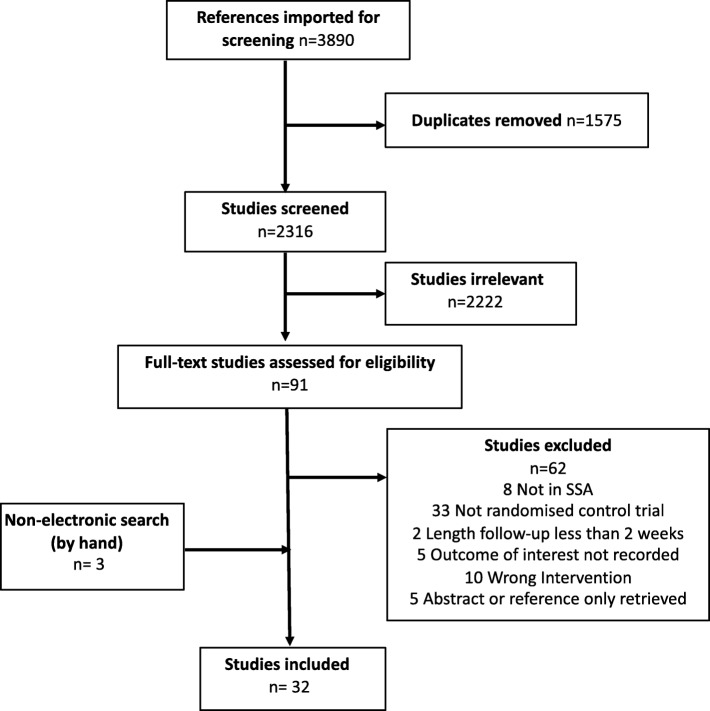
PRISMA flowchart of search results and study retrieval

Reports or abstracts of 2316 papers yielded 32 [23–54] studies of 2860 patients suitable for inclusion. Figure 1 outlines the flow of citations according to the PRISMA guidelines. Three international multi-centre studies were excluded because data were not available for SSA study participants, the number of SSA participants was less than 10% of the total trial population and the number of black Africans included was extremely low. There was a mix of studies across all regions of SSA although 53% were conducted in Nigeria or South Africa. The majority of studies were conducted over 2 decades ago with only 6 new papers in the last 10 years. They were also small in size, with a median number of 42 participants.

Additional file 2 details the study characteristics. In general, they were conducted in similar populations of middle-aged, black Africans from single urban centres. The mean age of participants was 51.2 years (SD 5.72), and 41.7% were males. Only one study [50] was conducted specifically in patients with other risk factors for cardiovascular disease. All major classes of antihypertensives were represented, and hydrochlorothiazide was the most common single agent.

None of our studies reported outcomes in terms of reduction in mortality or (cardiovascular) morbidity. All had some data on blood pressure-lowering efficacy, most reported in terms of office systolic or diastolic blood pressure. Two studies reported only ambulatory blood pressures [37, 52], and one study reported only mean arterial blood pressure change [25].

### Risk of bias and data quality

**Fig. 2:**
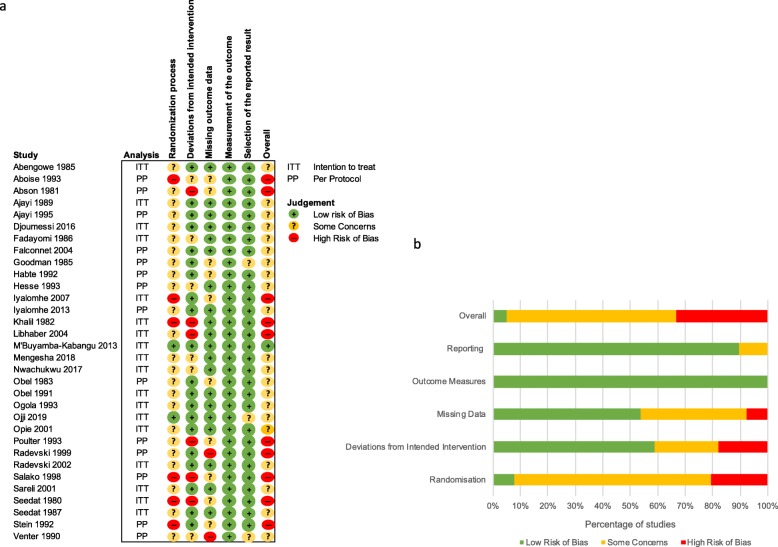
Quality assessment using Risk of Bias 2 tool. **a** Quality assessment of 32 different studies demonstrating low, high or some concerns of risk of bias. Five individual domains assessed as well as overall judgement. **b** Summary of the risk of bias assessments for all papers across 5 domains. ITT, intention to treat; PP, per protocol

Figure 2 summarises the quality assessment using the Risk of Bias 2 tool. Nearly all of our studies were at some or high risk of bias during randomisation, usually because of the lack of reporting on methods of randomisation, allocation or concealment to treatment. Fifty percent of our studies reported on a per-protocol basis raising concerns about deviations from the intended intervention. In general, reporting and analysis of outcomes w straightforward leading to low risk of reporting bias in most studies. Data were incomplete for 30 studies. For 23 studies, we were able to make reasonable approximations for missing information using the data provided at baseline and end time points. Five studies failed to report any measure of variance around quoted blood pressure measurements, and we used our imputation model as described above.

### Monotherapy

Twenty-six studies reported outcomes with single-agent versus placebo or monotherapy arms. In general, there were small numbers of participants and high levels of heterogeneity (overall *I*^2^ = 93.2%). One study [51] was a clear outlier, reporting a 60-mmHg average drop in BP with nifedipine monotherapy, and was excluded from the analysis as per previous reviews [19].

Overall pharmacotherapy caused a reduction of 8.51 mmHg in SBP (95% CI − 17.96, 0.94; *I*^2^ 67.2%) and 8.04 mmHg in DBP (95% CI − 4.97, − 11.12, *I*^2^ 0.0%) compared with placebo. The results between classes were mixed, with CCB the only class to show evidence for superiority in lowering both SBP and DBP. To explore this relationship further, we performed a network meta-analysis. Our network map (Table 1) showed that most information was available for diuretics versus other agents. There was a reasonable consistency of our model for comparing both SBP and DBP (global test for inconsistency 0.218 SBP and 0.531 DBP, network forests displayed in Additional file 3: Fig. S1). The only node to show inconsistency was ACEi and diuretics in SBP model (difference between direct and indirect evidence 18.61, *p* = 0.012), due to two small studies of less than ten patients in each arm [49, 54]. Table 1 shows most patients would be expected to have a good response to CCBs with a 18.46/11.64 drop in BP and 64% achieving BP control with monotherapy alone. Diuretics are also efficacious in lowering SBP and DBP but to a lesser magnitude*.* Conclusions about ACEi are limited by inconsistency and indirectness in the model but in general do not appear to be better than placebo when used alone. BB may reduce DBP but are no more effective than placebo in lowering SBP.

**Table 1:**
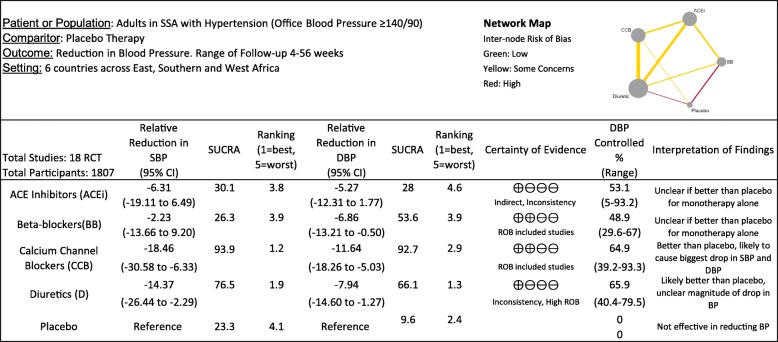
Network meta-analysis of monotherapy in SSA hypertensives

### Within class differences

Three studies investigated whether there are differences in the choice of CCB used. Sareli et al. [43] showed a 5/3-mmHg greater drop with nifedipine versus verapamil. Alternatively, when isradipine and nifedipine, two non-dihydropyridine agents, were compared, they were equally efficacious in lowering BP [23, 27].

### Combination therapy

There were only 3 studies of 207 patients which compared monotherapy with combination therapy. Although combination therapy showed a trend towards superiority with SBP/DBP (− 8.66; 95% CI − 18.72, 1.40/− 6.11; 95% CI − 9.15/− 3.07), the majority of this effect was from a small study of 31 participants comparing methyldopa with a beta-blocker and diuretic combination [33]. There was only 1 study which compared CCB monotherapy with combination therapy; here, perindopril plus hydrochlorothiazide was equally efficacious as high dose amlodipine (SBP − 1.65 mmHg in favour of ACEi + diuretic, 95% CI − 8.96, 5.66) [26].

**Fig. 3:**
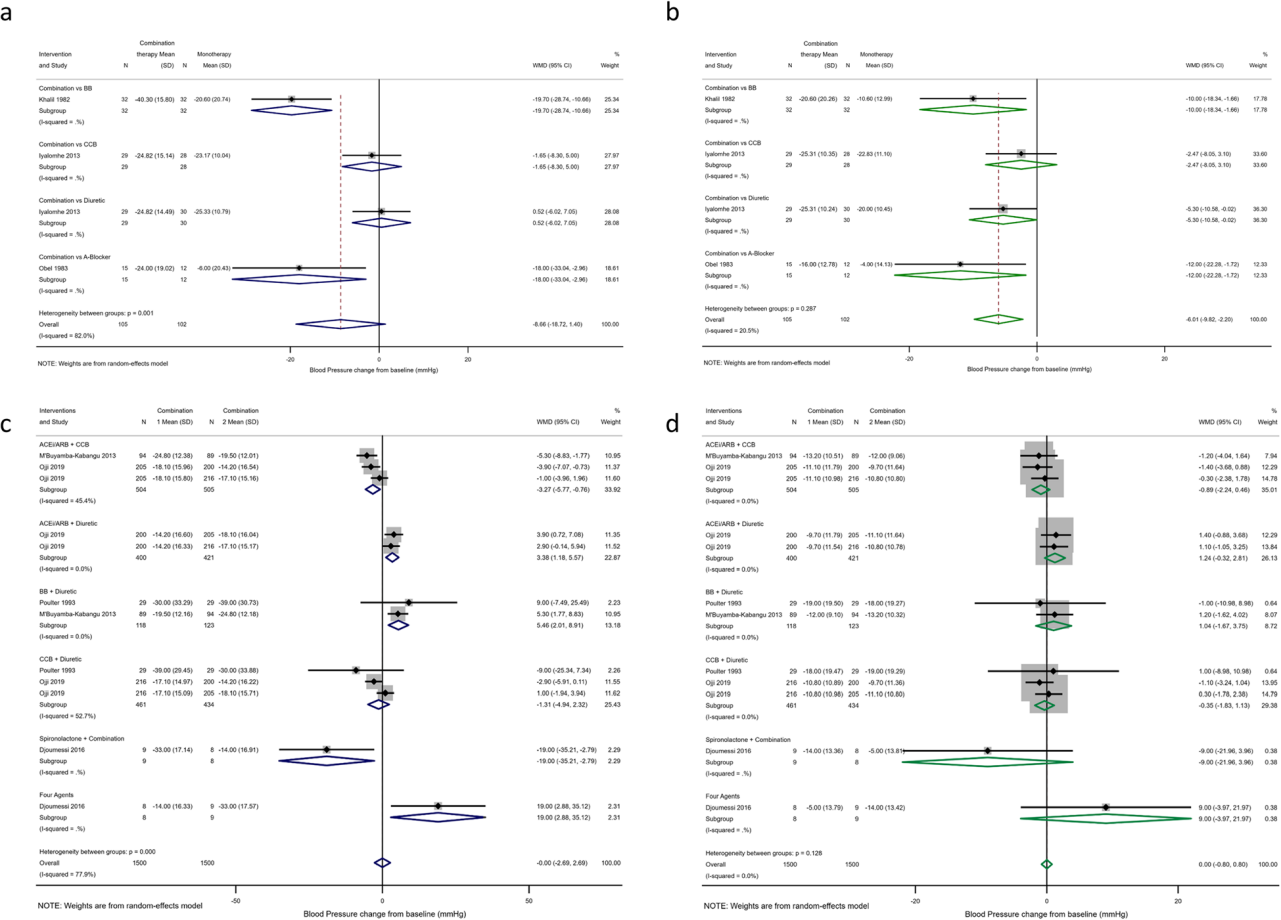
Meta-analysis of the changes in blood pressure with combination versus monotherapies (**a**, **b**) or different combination therapies (**c**, **d**). Combination versus monotherapy for change in systolic (**a**) and diastolic (**b**) blood pressures. Comparisons of different combination therapies for change in systolic (**c**) and diastolic (**d**) blood pressures

Four studies compared the different regimes of combination therapy. Djoumessi et al. [50] compared spironolactone as the fourth agent in resistant hypertension versus physician choice of alternative (alpha-blocker, ARB or BB) in Cameroon. There was a sizeably greater 19/9 mmHg drop in SBP/DBP with spironolactone, although the study was small in size. Three studies compared the combination of CCB, diuretics, ACEi or ARBs, and BBs including CREOLE, a recent large parallel RCT of combination therapy in SSA [37]. Our meta-analyses (Fig. 3) showed a small overall drop in blood pressure with ACEi/ARB + CCB although the absolute difference was small. There was no difference between diastolic blood pressures.

### Meta-regression and sensitivity analysis

We explored the potential sources of bias in our results through a meta-regression of the mean difference in treatment effect in each of our studies against publication year, sample population size, age and gender mix (Additional file 3: Fig. S2). There were no significant interactions, perhaps not surprising given the baseline similarities of the studies. Next, we explored the differences in the study design. Although most of our studies used DBP as an inclusion criterion, baseline SBP was not related to the mean difference in treatment effect size (coefficient 0.112, *p* = 0.345). The same was also true of dosing strategy, with no greater differences between the arms in trials which used titration to target BP rather than fixed dosing (coefficient 2.67, *p* = 0.162).

We performed a sensitivity analysis to determine how our imputed missing values may have affected our results. Excluding studies with missing standard deviations or other measures of variance did not change the conclusions of our meta-analysis (rankograms displayed in Additional file 3: Fig. S3) with CCBs still 78% most likely to be the best monotherapy agents. It did improve the consistency of our SBP model (global test for inconsistency *χ*^2^ = 6.96, *p* = 0.43). Although we found no association between the risk of bias and the effect size in our meta-analysis (coefficient 3.82, *p* = 0.39) when studies with high ROB were excluded from the network meta-analysis, this reduced the difference between CCB and diuretics (rank SBP 2.1, 1.6, *χ*^2^ = 2.74, *p* = 0.841; DBP 2.2, 2.2, *χ*^2^ = 2.56, *p* = 0.8611, respectively). Finally, a funnel plot of our meta-analysis raised no additional concerns of publication bias (Additional file 3: Fig. S4).

### Adverse events

Twenty-one (67.7%) studies reported any side effects of medication given. In most studies, this was not systematic, with only one study [37] adhering to the CONSORT guidelines [56]. Patients taking methyldopa experienced the highest level of side effects (77% of patients) although this was based on one study [33]. Of the major antihypertensive drug classes, fewest side effects were seen with beta-blockers (8%), although total patient numbers were smaller.

Table 2 displays the percentage of common side effects and serious adverse events noted for each drug class. Given reporting was often ad hoc, we avoided attributing ‘zero’ incidence to adverse effects not seen or calculating risk ratios. The main differences between classes were a greater occurrence of angioedema (1%) and cough (6%) with ACEi versus headache (4%) and ankle swelling (3%) with CCB. A total number of serious adverse events were small, but BBs were associated with more cardiac events than other classes. There were only two reported incidences of acute kidney injury, both occurring with ACE inhibitors. Of the three deaths which occurred across two studies, only one death due to cerebral haemorrhage whilst taking placebo therapy was clearly related to the trial arm (Table 2).

**Table 2:** Reported adverse events by therapeutic class

Class	Number	Withdrawn due to medication	Adverse effects reported (percentage of total participants)										
			Total (%)	Cough (%)	Headache (%)	Dizziness or postural hypotension (%)	Angioedema (%)	Ankle swelling (%)	Hypokalaemia (%)	Acute kidney injury (%)	Cardiac events (%)	Other serious events (%)	Death
ACEi or ARB													
Monotherapy	127	6	28.3	3.1	–	2.4	–	–	–	1.6	0.79	0.8	–
Total	638	24	17.2	5.2	0.9	1.4	0.9	1.6	0.9		0.14	0.1	–
Beta-blockers													
Monotherapy	135	0	8.1	–	–	2.2	–	–	–	–	1.21	–	–
Total	194	0	8.2	–	0.5	1.5	–	–	1.0	–	1.18	–	–
CCB													
Monotherapy	334	25	22.5	0.3	6.3	1.8	–	1.8	0.6	–	0.47	–	1*
Total	752	27	20.3	2.0	4.0	1.5	0.3	3.3	2.1	–	0.25	–	–
Diuretics													
Monotherapy	120	0	4.2	–	–	1.7	–	–	–	–	0.47	0.5	1^†^
Total	708	20	11.9	2.1	1.1	1.6	0.6	1.6	3.0	–	0.21	0.1	–
Other agents													
Methyldopa	13	1	76.9	–	–	–	–	–	–	–	–	–	–
Potassium	42	0	0.0	–	–	–	–	–	–	–	–	–	–
Placebo	39	0	0.0	–	–	–	–	–	–	–	–	–	1^~^
K+ sparing agents in combination	56	0	10.7	–	1.8	–	–	–	–	–	–	–	–

*K+* potassium

*On hydrochlorothiazide 12.5–25 mg and died from bowel obstruction

^†^On Verapamil 240–360 mg and died from pneumonia

^~^On placebo and died from cerebral haemorrhage, no increase in baseline blood pressure

^–^No adverse events, of this nature, reported

## Discussion

Our main findings were that overall pharmacotherapy lowered BP by 8.51/8.04 mmHg compared to placebo, and calcium channel blockers were the most effective single agent, with a relative − 17.96/0.94 mmHg reduction. Amongst CCBs, nifedipine was more effective than verapamil and showed similar effectiveness to isradipine. We found less efficacy of combination therapy compared to monotherapy than expected from studies elsewhere. However, this was likely due to the limited amount of data available for comparison. Sareli et al. [43] showed a clear additional reduction in blood pressure in non-controlled participants through combination therapy, independent of initial agent. In general, treatments were well-tolerated. The rates of angioedema were similar to previous reports in black patients, who are at greater risk compared with white populations [57, 58]. However, none of the data succeeded in exploring the relationship between treating blood pressure and reduction in primary or secondary cardiovascular disease. Taking this together, we can only draw conclusions on what treatments should be recommended in SSA to lower blood pressure, rather than to prevent meaningful patient outcomes such as stroke or cardiac disease.

The strengths of our review were that we conducted a comprehensive search across publication date, language and different pharmacological treatment strategies, rather than restricting results to a single class or a combination of agents. Through our meta-analyses, we have also summarised the data available, to give a more comprehensive picture of the efficacies of different treatment strategies. There are some limitations. Firstly, there is a significant publication barrier for resource-constrained institutions. We made a considered effort to include all studies regardless of age or size, including many which only exist in print version, and cross-referenced against other reviews of overlapping populations, but may have missed those never published or in non-peer review journals. Secondly, our search strategy specified that papers must have included some reference to Sub-Saharan Africa in the publication record. This may have missed large multi-centre studies which included African participants but did not reference this in the main text of the publication. However, in recent a global meta-analysis, just 11% of studies included SSA, and exclusively ACEi therapy in predominantly white South Africans [59]. Data reporting was incomplete, and we struggled to get responses from the authors (unsurprising given the era of many older publications). This has introduced some imprecision, with wide confidence intervals for the difference in effect sizes, especially in small studies. Despite this, our meta-regression showed there was a good consistency across reported results, and this was not affected by our imputation models. In addition, the number of studies and participants was too few for us to perform meaningful sub-group analyses, e.g. by period of publication, region of SSA or urban versus rural participants. Finally, as we have highlighted, there were concerns with the risk of bias, especially in randomisation to intervention. Our results were comparable across our sensitivity analysis implying broader generalisability across similar populations, but clearly modern trials are needed.

### Wider context

Akin to African-Americans, calcium channel blockers appear to be the most efficacious single agents in African patients. Vascular smooth muscle contractility in those of African descent is purportedly governed by higher concentrations of creatine kinase and lower nitric oxide, pertaining to dual targets for CCBs, which both decrease CK and increase nitrous oxide [12]. However, this has not been verified in SSA participants. Our finding of CCBs’ superiority is thus important in supporting consensus from both Pan-African guidance [11] and global public health initiatives [60].

Similarly, early initiation of combination therapy is a key pillar of the most recent hypertension guidelines [61, 62]. Advantages of combination therapy include tighter, earlier blood pressure control and reduction in dose-dependent side effects, plus augmentation of class effects. Addition of CCBs or diuretics can override the ineffectiveness of ACEi/ARBs in a low-renin/salt sensitivity phenotype, typical of those with African heritage [12]. Our results, predominantly based on the data from CREOLE, support using a CCB backbone combined with ACEi or thiazide diuretic. Policymakers may be keen to adopt a ‘magic bullet’ solution with a single top choice option for both mono and combination therapy aiding drug procurement and distribution as well as task-shifting in constrained health services. Whilst this is understandable, it would not take account of the heterogeneity in population risk factors and the potential benefits of drug classes outside of their direct effect on blood pressure. For example, none of the studies in our review included participants with co-existing cardiovascular or renal disease, patients in whom treatment with ACEi may be more appropriate than other blood pressure-lowering therapies.

There is an urgent need to undertake larger trials across regions and ethnicities in SSA, with the following specific questions. Foremost, we need evidence which therapy best addresses patient-centred outcomes like reduction in death and disability. Trials must be designed with attention to detail. Urban centres may be the locus of the current upsurge in hypertension cases. However, trials should not neglect rural communities, given the established burden of disease and distance to healthcare facilities. Closer observation of population heterogeneity may be fundamental to understanding why Africans are disproportionately affected by hypertension. Genetic polymorphisms which protect from common childhood infections may have pleiotropic effects later on, as illustrated with a variation of the APOL1 gene and trypanosomiasis infection [63]. Alternatively, the nature, severity and burden of infection, especially in early life, may play an important contribution [64]. Ageing HIV-infected populations are already at increased risk of stroke [65] and could have more vulnerable vasculature. Finally, there is a suggestion of a greater predisposition to hyperaldosteronism in some SSA populations, an important and treatable cause of resistant hypertension [66].

Pragmatically, it could be argued that these research priorities are less important than ensuring universal coverage of basic hypertension screening and simplistic treatment algorithms. The widespread success of HIV treatment programmes demonstrates the capability to address complexity within a public health approach [67, 68]. Innovative hypertension programmes are integrating care into existing HIV programmes [69], utilising mobile technology [70, 71] and implementing multi-pronged community interventions [72]. Harmonising these with knowledge of local population epidemiology and outcome of intervention could lead to a much greater understanding of how best to deliver care.

## Conclusion

Our work comprehensively summarises blood pressure trials in Sub-Saharan Africa over the last 50 years. Our findings support recommendations from recent guidelines and may be used to embed core principles, such as CCBs as first-line therapy, into local policy. Urgent expansion of research, which both addresses patient-centred outcomes and takes into account population diversity, is needed in order for SSA to meet global targets and curb the mounting crisis.

## Supplementary information

**Additional file 1.** Search Terms and strategy used for Ovid Medline search – January 2019. List of search terms used in Ovid Medline search performed in January 2019.

**Additional file 2.** Characteristics of Included Studies. Table summarising main characteristics of included studies, including data of publication, demographics of study population, and main outcomes.

**Additional file 3: **Supplementary Figures of additional results not displayed in the infographics of the main text. **Figure S1.** Forest Plots showing results of network meta-analysis of monotherapy on systolic and diastolic blood pressure. **Figure S2.** Trends in blood pressure lowering efficacy of treatment with (a) age, (b) gender and (c) number of participants and (d) year of publication. **Figure S3.** Rankograms for Network Meta-analysis with exclusion of imputed missing values (a) SBP (b) DBP and exclusion of studies rates as high risk of bias (c) SBP and (d) DBP. **Figure S4.** Funnel plot for global meta-analysis of 31 studies, grouped by blood pressure agent class.

## Data Availability

Summary data is available from open repository at Harvard Dataverse. Seeley, Anna, 2020, "Replication Data for: Systematic review and meta-analysis for hypertension treatment in Sub-Saharan Africa.", 10.7910/DVN/ZZRWOM, Harvard Dataverse, V1, UNF:6:Zwqu4pwKCQguybqu+tnjEw== [fileUNF].

## Abbreviations

ACEi: Angiotensin-converting enzyme inhibitor
ARB: Angiotensin receptor blocker
BNF: British National Formulary
BB: Beta-blocker
BP: Blood pressure
CCB: Calcium channel blocker
DBP: Diastolic blood pressure
HCT: Hydrochlorothiazide
HTN: Hypertension
ITT: Intention to treat
SBP: Systolic blood pressure
SD: Standard deviation
SSA: Sub-Saharan Africa
PP: Per protocol
PRISMA: Preferred Reporting Items for Systematic Reviews and Meta-Analyses
SR: Sustained release
WHO: World Health Organization
