# Supplementary material for: Pharmacotherapy for hypertension in Sub-Saharan Africa: a systematic review and network meta-analysis
Source: BMC Med. 2020 Mar 27;18:75. doi: 10.1186/s12916-020-01530-z (PMC7099775; doi:10.1186/s12916-020-01530-z)
Supplement: Supplementary file 1 — Additional file 1. Search Terms and strategy used for Ovid Medline search – January 2019. List of search terms used in Ovid Medline search performed in January 2019. [file 12916_2020_1530_MOESM1_ESM.docx]

**Additional File 1**

**Search Terms and strategy used for Ovid Medline search – January 2019**

| Search.Attempt.1.1 |
| --- |
|  |
| 1. Antihypertensive Agents/ |
| 2. Hypertension/ |
| 3. Angiotensin-Converting Enzyme Inhibitors/ |
| 4. CAPTOPRIL/ |
| 5. Enalapril/ |
| 6. FOSINOPRIL/ |
| 7. Imidapril.mp. [mp=title, abstract, original title, name of substance word, subject heading word, floating sub-heading word, keyword heading word, protocol supplementary concept word, rare disease supplementary concept word, unique identifier, synonyms] |
| 8. LISINOPRIL/ |
| 9. Moexipril.mp. [mp=title, abstract, original title, name of substance word, subject heading word, floating sub-heading word, keyword heading word, protocol supplementary concept word, rare disease supplementary concept word, unique identifier, synonyms] |
| 10. PERINDOPRIL/ |
| 11. Quinapril.mp. [mp=title, abstract, original title, name of substance word, subject heading word, floating sub-heading word, keyword heading word, protocol supplementary concept word, rare disease supplementary concept word, unique identifier, synonyms] |
| 12. RAMIPRIL/ |
| 13. Trandolapril.mp. [mp=title, abstract, original title, name of substance word, subject heading word, floating sub-heading word, keyword heading word, protocol supplementary concept word, rare disease supplementary concept word, unique identifier, synonyms] |
| 14. Aliskiren.mp. [mp=title, abstract, original title, name of substance word, subject heading word, floating sub-heading word, keyword heading word, protocol supplementary concept word, rare disease supplementary concept word, unique identifier, synonyms] |
| 15. 3 or 4 or 5 or 6 or 7 or 8 or 9 or 10 or 11 or 12 or 13 or 14 |
| 16. Angiotensin Receptor Antagonists/ |
| 17. Angiotensin II Type 1 Receptor Blockers/ |
| 18. Azilsartan.mp. [mp=title, abstract, original title, name of substance word, subject heading word, floating sub-heading word, keyword heading word, protocol supplementary concept word, rare disease supplementary concept word, unique identifier, synonyms] |
| 19. Candesartan.mp. [mp=title, abstract, original title, name of substance word, subject heading word, floating sub-heading word, keyword heading word, protocol supplementary concept word, rare disease supplementary concept word, unique identifier, synonyms] |
| 20. TETRAZOLES/ |
| 21. Eprosartan.mp. [mp=title, abstract, original title, name of substance word, subject heading word, floating sub-heading word, keyword heading word, protocol supplementary concept word, rare disease supplementary concept word, unique identifier, synonyms] |
| 22. Irbesartan.mp. [mp=title, abstract, original title, name of substance word, subject heading word, floating sub-heading word, keyword heading word, protocol supplementary concept word, rare disease supplementary concept word, unique identifier, synonyms] |
| 23. LOSARTAN/ |
| 24. Olmesartan.mp. [mp=title, abstract, original title, name of substance word, subject heading word, floating sub-heading word, keyword heading word, protocol supplementary concept word, rare disease supplementary concept word, unique identifier, synonyms] |
| 25. Telmisartan.mp. [mp=title, abstract, original title, name of substance word, subject heading word, floating sub-heading word, keyword heading word, protocol supplementary concept word, rare disease supplementary concept word, unique identifier, synonyms] |
| 26. VALSARTAN/ |
| 27. 16 or 17 or 18 or 19 or 20 or 21 or 22 or 23 or 24 or 25 or 26 |
| 28. Adrenergic beta-Antagonists/ |
| 29. ACEBUTOLOL/ |
| 30. ATENOLOL/ |
| 31. BISOPROLOL/ |
| 32. Carvedilol.mp. [mp=title, abstract, original title, name of substance word, subject heading word, floating sub-heading word, keyword heading word, protocol supplementary concept word, rare disease supplementary concept word, unique identifier, synonyms] |
| 33. CELIPROLOL/ |
| 34. Esmolol.mp. [mp=title, abstract, original title, name of substance word, subject heading word, floating sub-heading word, keyword heading word, protocol supplementary concept word, rare disease supplementary concept word, unique identifier, synonyms] |
| 35. LABETALOL/ |
| 36. METOPROLOL/ |
| 37. NADOLOL/ |
| 38. NEBIVOLOL/ |
| 39. OXPRENOLOL/ |
| 40. PINDOLOL/ |
| 41. PROPRANOLOL/ |
| 42. SOTALOL/ |
| 43. TIMOLOL/ |
| 44. 28 or 29 or 30 or 31 or 32 or 33 or 34 or 35 or 36 or 37 or 38 or 39 or 40 or 41 or 42 or 43 |
| 45. Calcium Channel Blockers/ |
| 46. AMLODIPINE/ |
| 47. DILTIAZEM/ |
| 48. FELODIPINE/ |
| 49. Lacidipine.mp. [mp=title, abstract, original title, name of substance word, subject heading word, floating sub-heading word, keyword heading word, protocol supplementary concept word, rare disease supplementary concept word, unique identifier, synonyms] |
| 50. Lercanidipine.mp. [mp=title, abstract, original title, name of substance word, subject heading word, floating sub-heading word, keyword heading word, protocol supplementary concept word, rare disease supplementary concept word, unique identifier, synonyms] |
| 51. NICARDIPINE/ |
| 52. NIFEDIPINE/ |
| 53. NIMODIPINE/ |
| 54. VERAPAMIL/ |
| 55. 45 or 46 or 47 or 48 or 49 or 50 or 51 or 52 or 53 or 54 |
| 56. Diuretics, Potassium Sparing/ |
| 57. AMILORIDE/ |
| 58. BUMETANIDE/ |
| 59. Eplerenone.mp. [mp=title, abstract, original title, name of substance word, subject heading word, floating sub-heading word, keyword heading word, protocol supplementary concept word, rare disease supplementary concept word, unique identifier, synonyms] |
| 60. FUROSEMIDE/ |
| 61. SPIRONOLACTONE/ |
| 62. Torasemide.mp. [mp=title, abstract, original title, name of substance word, subject heading word, floating sub-heading word, keyword heading word, protocol supplementary concept word, rare disease supplementary concept word, unique identifier, synonyms] |
| 63. Triamterene.mp. [mp=title, abstract, original title, name of substance word, subject heading word, floating sub-heading word, keyword heading word, protocol supplementary concept word, rare disease supplementary concept word, unique identifier, synonyms] |
| 64. DIURETICS/ |
| 65. Sodium Chloride Symporter Inhibitors/ |
| 66. Sodium Potassium Chloride Symporter Inhibitors/ |
| 67. BENDROFLUMETHIAZIDE/ |
| 68. Co-amilozide.mp. [mp=title, abstract, original title, name of substance word, subject heading word, floating sub-heading word, keyword heading word, protocol supplementary concept word, rare disease supplementary concept word, unique identifier, synonyms] |
| 69. HYDROCHLOROTHIAZIDE/ |
| 70. INDAPAMIDE/ |
| 71. Chlorthalidone/ |
| 72. CYCLOPENTHIAZIDE/ |
| 73. METOLAZONE/ |
| 74. XIPAMIDE/ |
| 75. THIAZIDES/ |
| 76. 56 or 57 or 58 or 59 or 60 or 61 or 62 or 63 or 64 or 65 or 66 or 67 or 68 or 69 or 70 or 71 or 72 or 73 or 74 or 75 |
| 77. Adrenergic alpha-Antagonists/ |
| 78. Alfuzosin.mp. [mp=title, abstract, original title, name of substance word, subject heading word, floating sub-heading word, keyword heading word, protocol supplementary concept word, rare disease supplementary concept word, unique identifier, synonyms] |
| 79. DOXAZOSIN/ |
| 80. INDORAMIN/ |
| 81. PRAZOSIN/ |
| 82. Tamsulosin.mp. [mp=title, abstract, original title, name of substance word, subject heading word, floating sub-heading word, keyword heading word, protocol supplementary concept word, rare disease supplementary concept word, unique identifier, synonyms] |
| 83. Terazosin.mp. [mp=title, abstract, original title, name of substance word, subject heading word, floating sub-heading word, keyword heading word, protocol supplementary concept word, rare disease supplementary concept word, unique identifier, synonyms] |
| 84. 77 or 78 or 79 or 80 or 81 or 82 or 83 |
| 85. METHYLDOPA/ |
| 86. CLONIDINE/ |
| 87. Moxonidine.mp. [mp=title, abstract, original title, name of substance word, subject heading word, floating sub-heading word, keyword heading word, protocol supplementary concept word, rare disease supplementary concept word, unique identifier, synonyms] |
| 88. HYDRALAZINE/ |
| 89. MINOXIDIL/ |
| 90. Phenoxybenzamine/ |
| 91. Phentolamine/ |
| 92. Nitroprusside/ |
| 93. Guanethidine/ |
| 94. Vasodilator Agents/ |
| 95. 85 or 86 or 87 or 88 or 89 or 90 or 91 or 92 or 93 or 94 |
| 96. 1 or 15 or 27 or 44 or 55 or 76 or 95 |
| 97. 2 and 96 |
| 98. ANGOLA/ |
| 99. BENIN/ |
| 100. BOTSWANA/ |
| 101. Burkina Faso/ |
| 102. Upper Volta.mp. |
| 103. BURUNDI/ |
| 104. CAMEROON/ |
| 105. Cabo Verde/ |
| 106. Central African Republic/ |
| 107. CHAD/ |
| 108. COMOROS/ |
| 109. CONGO/ |
| 110. Cote d'Ivoire/ |
| 111. Ivory Coast.mp. |
| 112. Zaire.mp. or "Democratic Republic of the Congo"/ |
| 113. French Somaliland.mp. or Djibouti/ |
| 114. Equatorial Guinea/ |
| 115. ERITREA/ |
| 116. ETHIOPIA/ |
| 117. Gabonese Republic.mp. or Gabon/ |
| 118. GAMBIA/ |
| 119. Gold Coast.mp. or Ghana/ |
| 120. GUINEA/ |
| 121. Guinea-Bissau/ |
| 122. KENYA/ |
| 123. Basutoland.mp. or Lesotho/ |
| 124. LIBERIA/ |
| 125. Malagasy Republic.mp. or Madagascar/ |
| 126. Nyasaland.mp. or Malawi/ |
| 127. MALI/ |
| 128. MAURITANIA/ |
| 129. MAURITIUS/ |
| 130. Mayotte.mp. [mp=title, abstract, original title, name of substance word, subject heading word, floating sub-heading word, keyword heading word, protocol supplementary concept word, rare disease supplementary concept word, unique identifier, synonyms] |
| 131. MOZAMBIQUE/ |
| 132. NAMIBIA/ |
| 133. NIGER/ |
| 134. NIGERIA/ |
| 135. Reunion/ |
| 136. Rwanda/ or Ruanda-Urundi.mp. |
| 137. Sao Tome & Principe.mp. [mp=title, abstract, original title, name of substance word, subject heading word, floating sub-heading word, keyword heading word, protocol supplementary concept word, rare disease supplementary concept word, unique identifier, synonyms] |
| 138. Sao Tome.mp. [mp=title, abstract, original title, name of substance word, subject heading word, floating sub-heading word, keyword heading word, protocol supplementary concept word, rare disease supplementary concept word, unique identifier, synonyms] |
| 139. SENEGAL/ |
| 140. SEYCHELLES/ |
| 141. Sierra Leone/ |
| 142. SOMALIA/ |
| 143. South Africa/ |
| 144. SOUTH SUDAN/ or SUDAN/ |
| 145. SWAZILAND/ |
| 146. Togolese Republic.mp. or Togo/ |
| 147. UGANDA/ |
| 148. United Republic of Tanzania.mp. or Tanzania/ |
| 149. ZAMBIA/ |
| 150. Zimbabwe/ or Rhodesia.mp. |
| 151. 98 or 99 or 100 or 101 or 102 or 103 or 104 or 105 or 106 or 107 or 108 or 109 or 110 or 111 or 112 or 113 or 114 or 115 or 116 or 117 or 118 or 119 or 120 or 121 or 122 or 123 or 124 or 125 or 126 or 127 or 128 or 129 or 130 or 131 or 132 or 133 or 134 or 135 or 136 or 137 or 138 or 139 or 140 or 141 or 142 or 143 or 144 or 145 or 146 or 147 or 148 or 149 or 150 |
| 152. "Africa South of the Sahara"/ |
| 153. "Subsaharan Africa".mp. |
| 154. Africa, Eastern/ |
| 155. Africa, Southern/ |
| 156. Africa, Western/ |
| 157. 151 or 152 or 153 or 154 or 155 or 156 |
| 158. ((high or elevat$ or rais$) adj2 blood pressure).mp. [mp=title, abstract, original title, name of substance word, subject heading word, floating sub-heading word, keyword heading word, protocol supplementary concept word, rare disease supplementary concept word, unique identifier, synonyms] |
| 159. 2 or 158 |
| 160. 96 and 157 and 159 |
