# Supplementary material for: Pharmacotherapy for hypertension in Sub-Saharan Africa: a systematic review and network meta-analysis
Source: BMC Med. 2020 Mar 27;18:75. doi: 10.1186/s12916-020-01530-z (PMC7099775; doi:10.1186/s12916-020-01530-z)
Supplement: Supplementary file 2 — Additional file 2. Characteristics of Included Studies. Table summarising main characteristics of included studies, including data of publication, demographics of study population, and main outcomes. [file 12916_2020_1530_MOESM2_ESM.docx]

**Additional File 2 - Characteristics of Included Studies**

| **Study** | **Country** | **Centre** | **BP Inclusion Criteria** | **Age (yrs)** | **Comorbidity** | **Total N** | **Black Ethnicity (%)** | **Male (%)** | **Trial Design** | **Agent 1** | **Daily Dose** | **N** | **Agent 2** | **Daily Dose** | **N** | **Primary Outcome** |
| --- | --- | --- | --- | --- | --- | --- | --- | --- | --- | --- | --- | --- | --- | --- | --- | --- |
| **Abengowe 1985** | Nigeria | Single, urban | DBP 95-120 | 25-66 | No | 45 | 100 | 40 | Parallel RCT | Acebutolol | 400 - 800mg | 27 | Propranolol | 160-320mg | 18 | Change in BP |
| **Aboise 1993** | Nigeria | Single, urban | DBP 95-140 | Adults | No | 42 | n/a | 39 | Cross-over RCT | Isradipine | 5-10mg | 16 | Nifedipine | 40-80mg | 14 | Change in BP |
| **Abson 1981** | Zimbabwe | Not stated | DBP 100-120 | 23-65 | No | 36 | 100 | 33 | Cross-over RCT | Atenolol | 100mg | 23 | Atenolol | 200mg | 23 | Change in BP |
|  |  |  |  |  |  |  |  |  |  | Placebo | n/a | 23 |  |  |  |  |
| **Ajayi 1989** | Nigeria | Not stated | DBP >95 | 30-75 | No | 20 | 100* | 45 | Parallel RCT | Enalapril | 20mg | 10 | HCT | 50mg | 10 | Change in BP |
| **Ajayi 1995** | Nigeria | Not stated | DBP ≥ 95 | 45-63 | No | 19 | 100* | 47.4 | Parallel RCT | Amlodipine | 5-10mg | 10 | HCT | 25-50mg | 9 | Change in BP |
| **Djoumessi 2016** | Cameroon | Single, urban | Office BP ≥140/90 or self-measured ≥130/80 on ≥3 agents | Adults ≤75 | T2DM | 17 | 100* | 47.1 | Parallel RCT | Spironolactone | 25mg | 9 | Other agent (candersartan atenolol  methyldopa) | 100mg or 100mg or 750mg | 8 | Change in BP |
| **Fadayomi 1986** | Nigeria | Not stated | DBP ≥ 110 in new HTN or ≥100 on treatment | 37-59 | No | 32 | 100* | 56.3 | Parallel RCT | Nifedipine | 40mg | 16 | Placebo | n/a | 16 | Change in BP |
| **Falconnet 2004** | Seychelles | Single, mixed | Amb BP >140/90 | 43-56 | No | 61 | 100 | 53.7 | Cross-over RCT | Lisinopril | 20mg | 52 | HCT | 25mg | 52 | Change in BP |
| **Goodman 1985** | South Africa | Single, urban | DBP 90-120 | 18-60 | No | 26 | 100 | n/a | Parallel RCT | Enalapril | 10mg | 13 | Propranolol | 80mg | 13 | Change in BP |
|  |  |  |  |  |  |  |  |  |  | Enalapril + HCT | 25-50mg | 13 | Propranolol + HCT | 25-50mg | 13 |  |
| **Habte 1992** | Ethiopia | Single, urban | DBP 95-120 after 2 weeks salt restriction | 26-62 | No | 26 | 100* | 50 | Parallel RCT | HCT | 25-50mg | 9 | Timolol | 10-20mg | 10 | Change in BP |
|  |  |  |  |  |  |  |  |  |  | Enalapril | 10-20mg | 7 |  |  |  |  |
| **Hesse 1993** | Ghana | Not stated | DBP 95-114 | 25-70 | No | 70 | 100 | 40 | Parallel RCT | Isradipine | 5mg | 40 | Nifedipine | 30mg | 30 | Change in BP |
| **Iyalomhe 2007** | Nigeria | Single, urban | BP > 160/95 and <180/110 | 32-80 | No | 80 | 100* | 50 | Parallel RCT | HCT | 25mg | 20 | Furosemide | 40mg | 20 | Change in BP |
| **Iyalomhe 2013** | Nigeria | Multiple, urban | BP >160/90 and ≤180/120 | 31-86 | No | 90 | 100* | 50 | Factorial | Amlodipine | 5mg | 28 | HCT | 25mg | 30 | Change in BP |
|  |  |  |  |  |  |  |  |  |  | Amlodipine + HCT | 5mg + 25mg | 29 |  |  |  |  |
| **Khalil 1982** | Sudan | Single, urban | BP ≥ 140/90 | 35-67 | 58% complicated HTN | 38 | 100* | n/a | Cross-over RCT | Acebutolol | 400mg | 32 | Acebutolol + HCT/amiloride | 400mg + 50mg/5mg | 32 | Change in BP |
| **Libhaber 2004** | South Africa | Single, urban | DBP ≥90 and ≤114 | 21-70 | No | 125 | 100* | 32 | Parallel RCT | Indapamide | 1.5mg | 61 | Amlodipine | 5mg | 64 | Change in BP and LV wall depth |
|  |  |  |  |  |  |  |  |  |  | Indapamide ± Peridonpril | 1.5mg ± 4mg | 61 | Amlodipine | 5-10mg | 64 |  |
| **M’Buyamba-Kabangu 2013 (NOAAH)** | 7 SSA Centre | Multiple, mixed | SBP 140-179 and or DBP 90-109 | 30-69 | No | 183 | 100 | 48 | Parallel RCT | HCT + bisoprolol | 6.25mg + 5-10mg | 89 | Amlodipine + Valsartan | 5-10mg + 160mg | 94 | Change in BP |
| **Mengesha 2018** | Ethiopia | Multiple, mixed | BP ≥ 140/90 | Adults | No | 141 | 100* | 39.7 | Parallel RCT | Enalapril | 5-15mg | 44 | HCT | 12.5mg-25mg | 44 | Change in BP |
|  |  |  |  |  |  |  |  |  |  | Nifedipine | 20-40mg | 44 |  |  |  |  |
| **Nwachukwu 2017** | Nigeria | Single, urban | SBP 140-179 and or DBP 90-109 | 33-60 | No | 50 | 100* | 56 | Parallel RCT | Amlodipine | 5mg | 25 | HCT | 25mg | 25 | Change in BP |
| **Obel 1983** | Kenya | Single, urban | DBP 95-120 | 21-60 | No | 31 | 96.7 | 34.4 | Parallel RCT | Timolol + HCT + amiloride (Moducren) | 1- 2 tablets (10mg + 25mg + 2.5mg) | 12 | Methyldopa | 750-3000mg | 15 | Change in BP |
| **Obel 1991** | Kenya | Single, urban | DBP 105-110 | 21-60 | No | 84 | 100 | 52.4 | Parallel RCT | Potassium Supplement | 64mmol | 42 | Bendrofluazide | 10mg | 42 | Change in BP |
| **Ogola 1993** | Kenya | Single, urban | DBP 100-110 | 18-65 | No | 60 | 100 |  | Parallel RCT | Hydroflumethazide | 50mg | 30 | Propranolol | 80-160mg | 30 | Change in BP |
| **Ojji 2019 (CREOLE)** | 6 SSA centres | Multiple, mixed | SBP 150-179 untreated or SBP 140-159 on 1 agent | 30-79 | No | 621 | 100 | 36.7 | Factorial | Amlodipine + HCT | 5-10mg +12.5-25mg | 216 | Amlodipine + Perinopril | 5-10mg + 4-8mg | 205 | Change in BP |
|  |  |  |  |  |  |  |  |  |  | Perinopril + HCT | 48 mg+ 12.5-25mg | 200 |  |  |  |  |
| **Opie 2001 (ANCHOR)** | South Africa | Multiple, mixed | DBP 95-114 | 18-75 | No | 206 | 30 | 45.2 | Parallel RCT | Nisoldipine | 10mg | 49 | Nisoldipine | 20mg | 51 | Change in BP |
|  |  |  |  |  |  |  |  |  |  | Nisoldipine | 30mg | 48 | Placebo | n/a | 58 |  |
| **Poulter 1993** | Kenya | Single, urban | DBP > 90 after 4 weeks on HCT | 30-69 | No | 37 | 100 | n/a | Cross-over RCT | HCT + Nifedipine | 50mg + 40mg | 29 | HCT + Propranolol | 50 + 160mg | 29 | Change in BP |
| **Radevski 1999** | South Africa | Single, urban | Amb DBP ≥110 and ≤ 140 | 38-58 | No | 143 | 100 | 47.1 | Parallel RCT | Nisoldipine | 10-40mg | 53 | Enalapril | 10-40mg | 43 | Change in BP and LV wall depth |
| **Radevski 2002** | South Africa | Single, urban | DBP 95-114 + Amb DBP 90-109 | Adults | No | 42 | 100 | n/a | Parallel RCT | Indapamide | 2.5mg | 20 | HCT | 12.5mg | 22 | Change in BP |
| **Salako 1998** | Nigeria | Single, urban | DBP 95-114 | 35-71 | No | 41 | 100* | 31.7 | Parallel RCT | Lacidipine | 4-6mg | 24 | HCT | 25-50mg | 17 | Change in BP |
| **Sareli 2001** | South Africa | Single, urban | Amb DBP 90-114 | 18-70 | No | 409 | 100 | 23 | Parallel RCT | Nifedipine GI | 30-60mg | 233 | Verapamil SR | 240-360mg | 58 | Change in BP and LV wall depth |
|  |  |  |  |  |  |  |  |  |  | HCT | 12.5-25mg | 58 | Enalapril | 10-20mg | 60 |  |
| **Seedat 1980** | South Africa | Single, urban | DBP 100-115 | Adults | No | 24 | 100 | n/a | Cross-over RCT | Atenolol | 100mg | 24 | Chlor-thalidone | 25mg | 24 | Change in BP |
|  |  |  |  |  |  |  |  |  |  | Atenolol + Chlorthalidone | 25mg+ 100mg | 24 |  |  |  |  |
| **Seedat 1987** | South Africa | Single, urban | DBP 91-115 | Adults | 47% LVH | 36 | 53 | 33.3 | Parallel RCT | Atenolol | 50-200mg | 12 | Lisinopril | 20-80mg | 24 | Change in BP |
|  |  |  |  |  |  |  |  |  |  | Atenolol + HCT | 175mg + 11mg | 12 | Lisinopril + HCT | 71 + 15mg | 24 |  |
| **Stein 1992** | Zimbabwe | Single, urban | DBP 96-115 | <70 | No | 25 | 100 | 52.6 | Cross-over RCT | HCT | 6.25-50mg | 19 | Placebo | n/a | 19 | Change in BP |
| **Venter 1990** | South Africa | Single, urban | DBP 95-115 | 25-65 | No | 56 | 100 | n/a | Cross-over RCT | Penbutolol | 40-80mg | 35 | Placebo | n/a | 35 | Change in BP |

* implied from text in paper, not explicitly given † As Combined tablet 'Tenoretic'

Abbreviations: Ambulatory blood pressure (amp), hypertension (HTN), hydrochlorothiazide (HCT), left ventricular (LV), randomised control trial (RCT), sustained release (SR)
