# Supplementary material for: Pharmacotherapy for hypertension in Sub-Saharan Africa: a systematic review and network meta-analysis
Source: BMC Med. 2020 Mar 27;18:75. doi: 10.1186/s12916-020-01530-z (PMC7099775; doi:10.1186/s12916-020-01530-z)

**Additional File 3: Figure S1**

Title: Forest Plots showing results of network meta-analysis of monotherapy for (a) systolic and (b) diastolic blood pressure lowering effect.


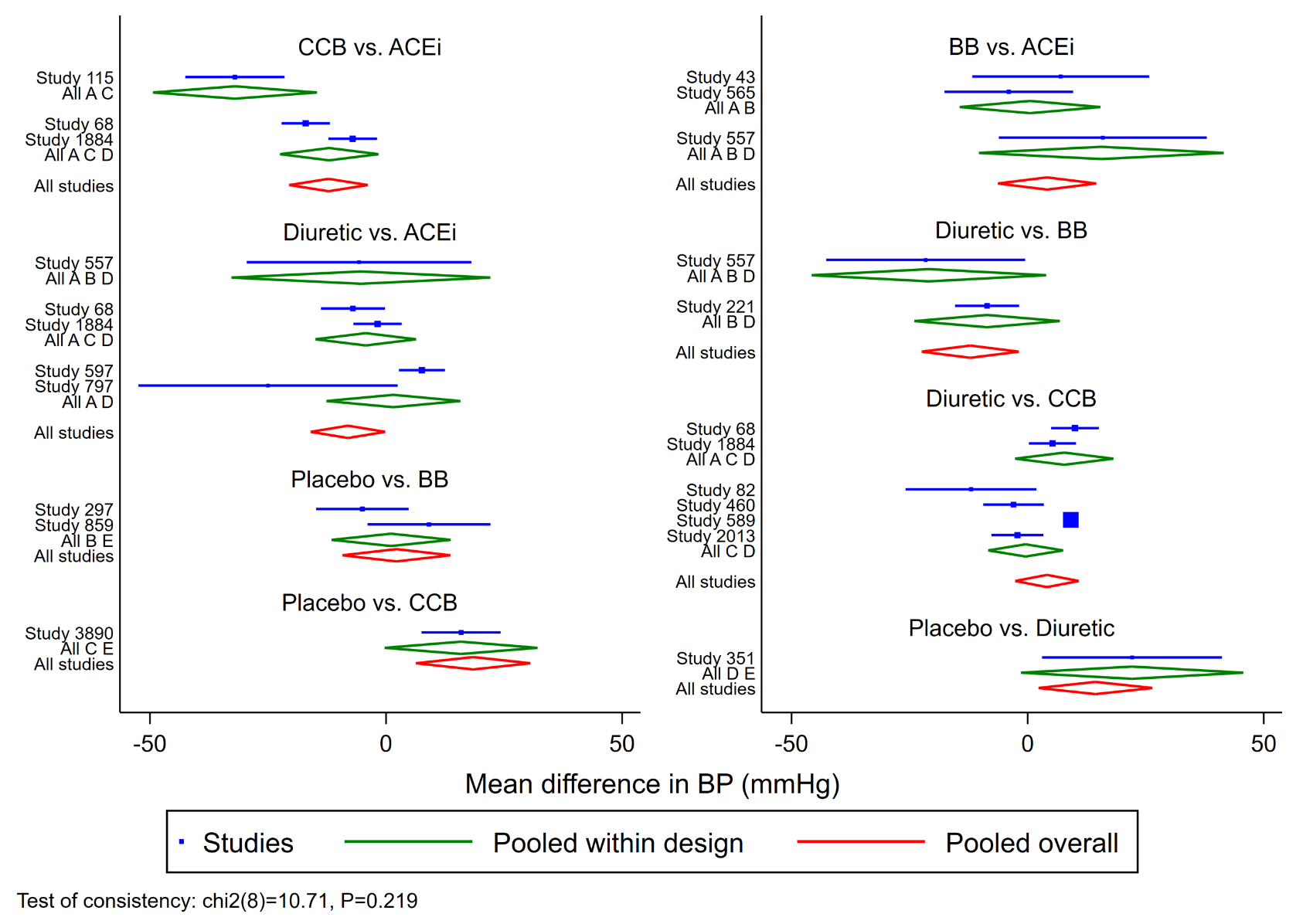


a


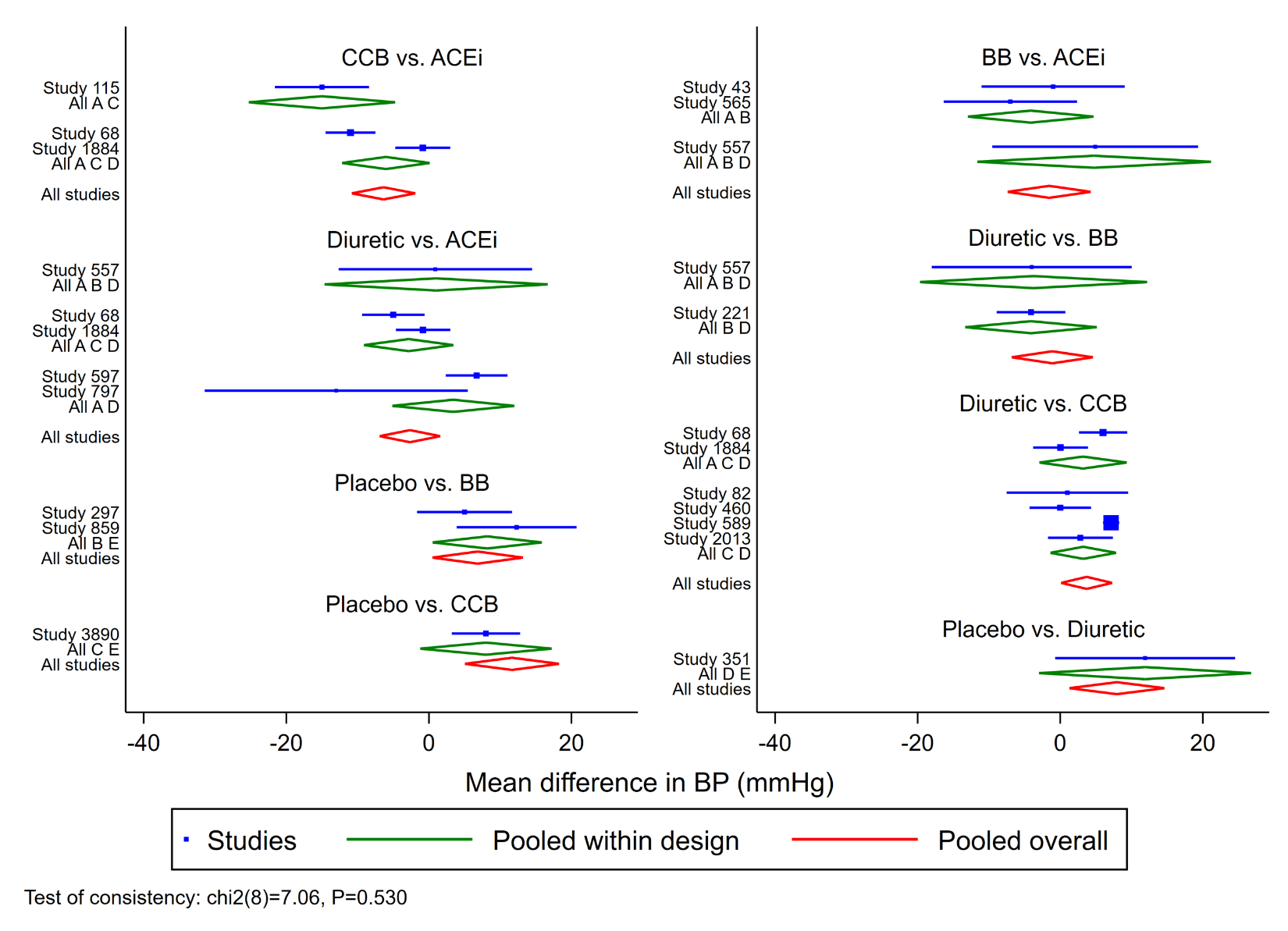


b

**Additional File 3: Figure S2**

Title: Trends in blood pressure lowering efficacy of treatment with (a) age, (b) gender and (c) number of participants as well as (d) year of publication

a

**
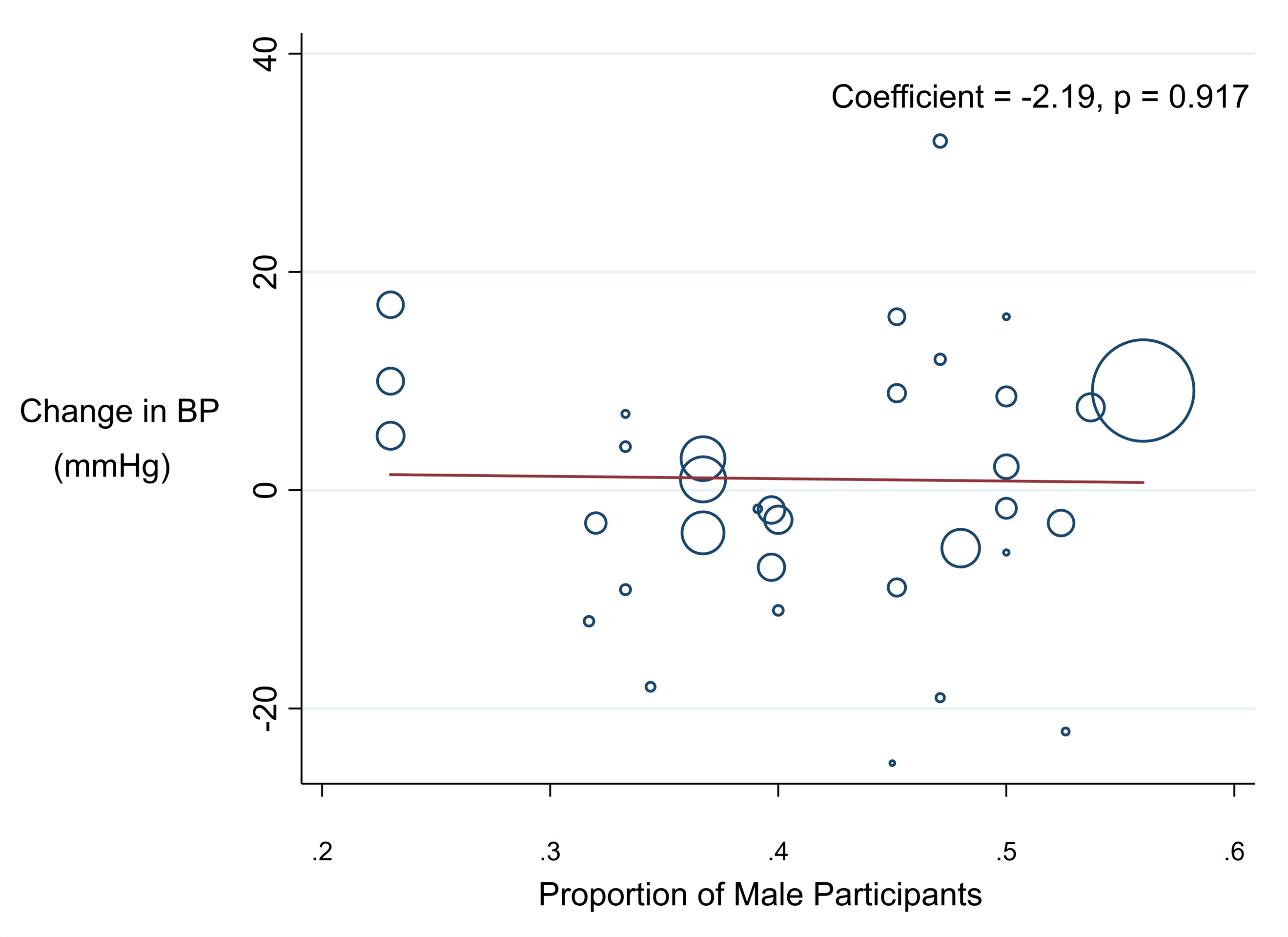
**

b

**
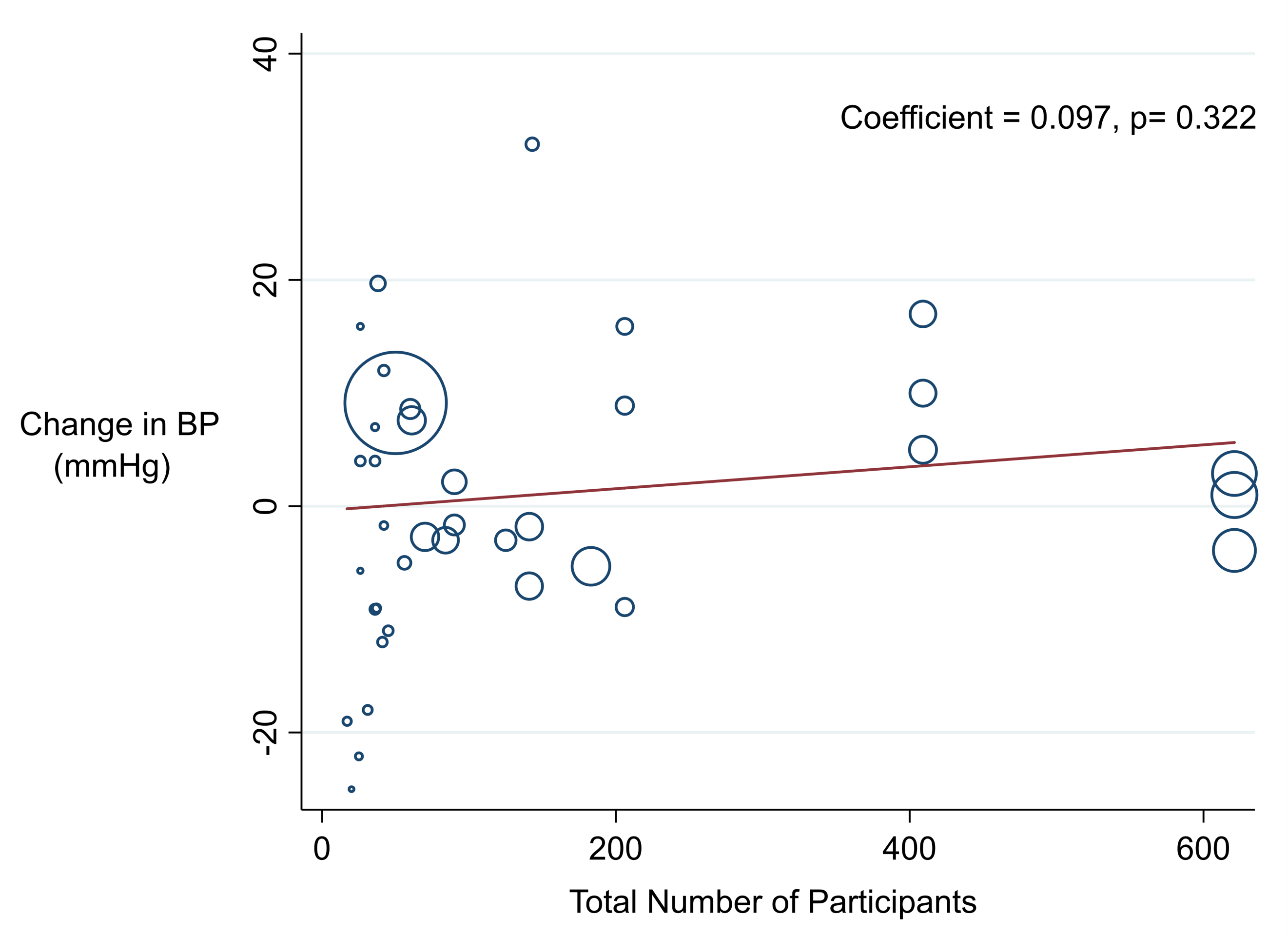
**

c

**
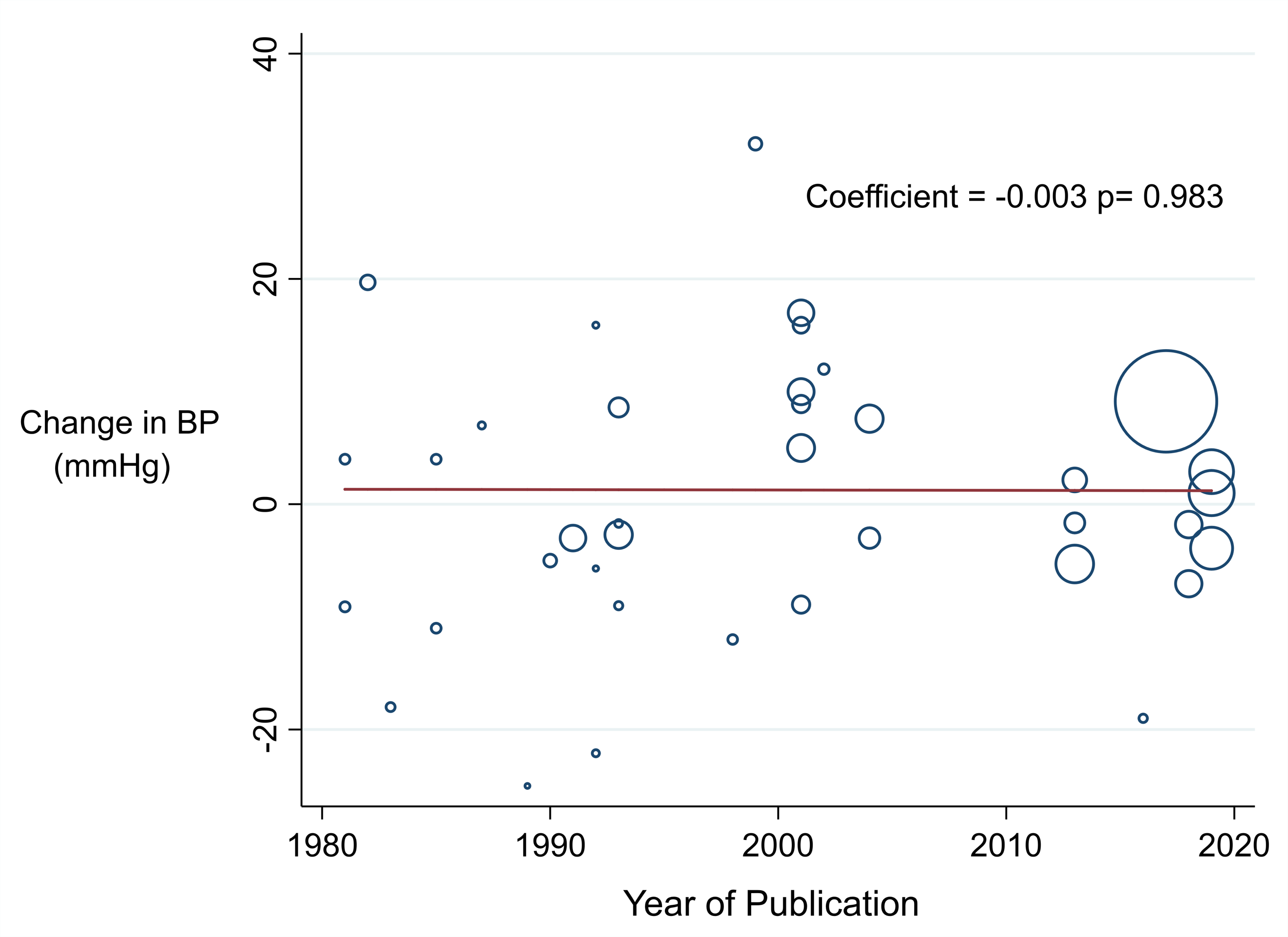
**

d

**Additional File 3: Figure S3**

Title; Rankograms for Network Meta-analysis with exclusion of imputed missing values (a) SBP (b) DBP and exclusion of studies rates as high risk of bias (c) SBP and (d) DBP. For each plot tests of consistency given for overall network meta-analysis as well as surface under cumulative ranking curve area (SUCRA) and relative mean rank (1 best 🡪 5 worst) compared to other treatments.

a


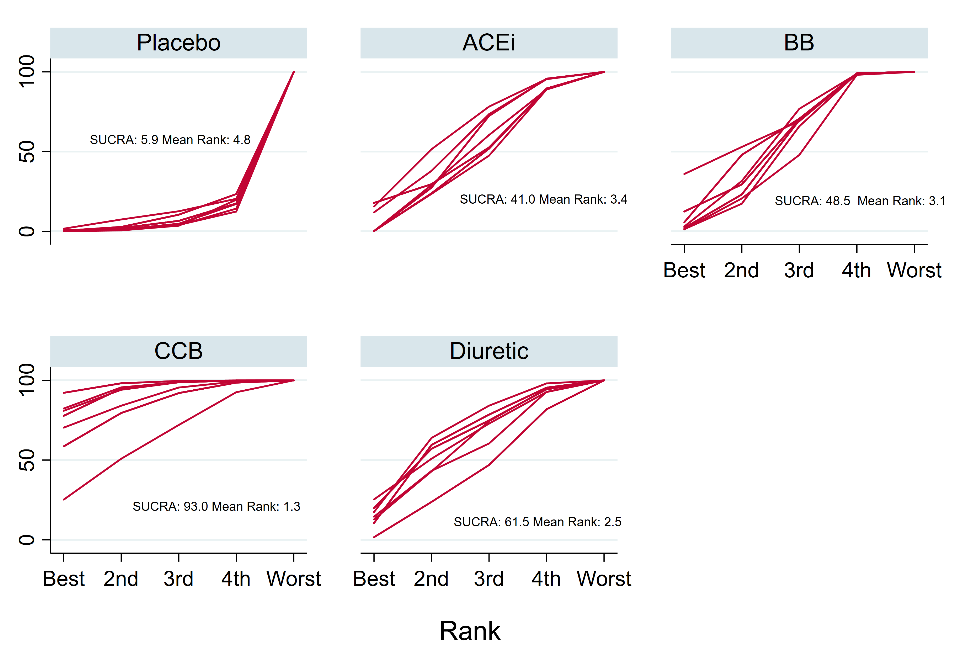


Test of consistency

chi^2^ =7.22

p = 0.407

b

c


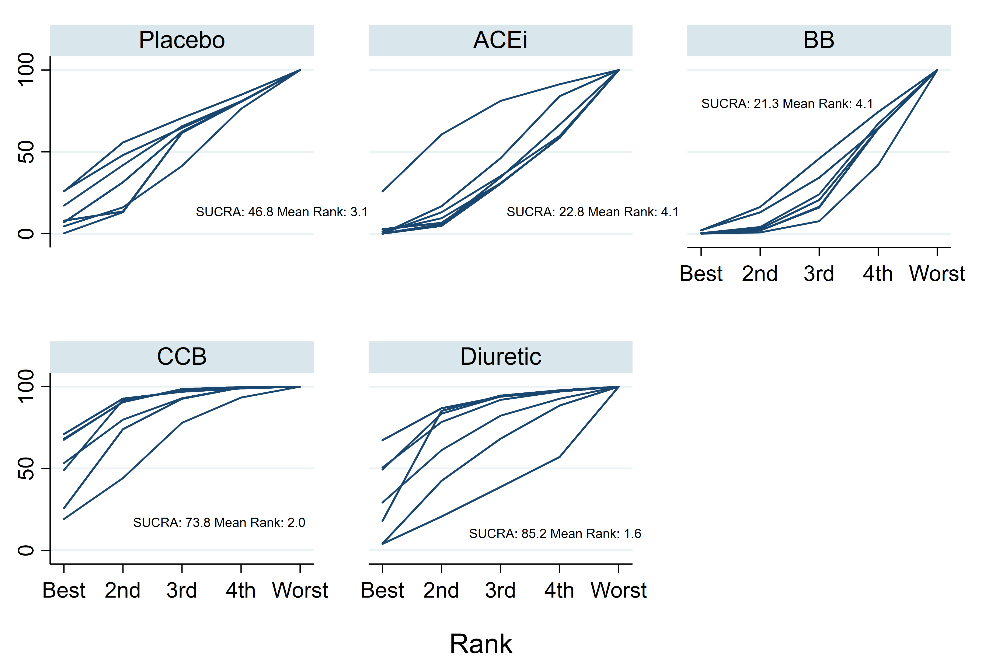


Test of consistency

chi^2^ =2.74

p = 0.84

d


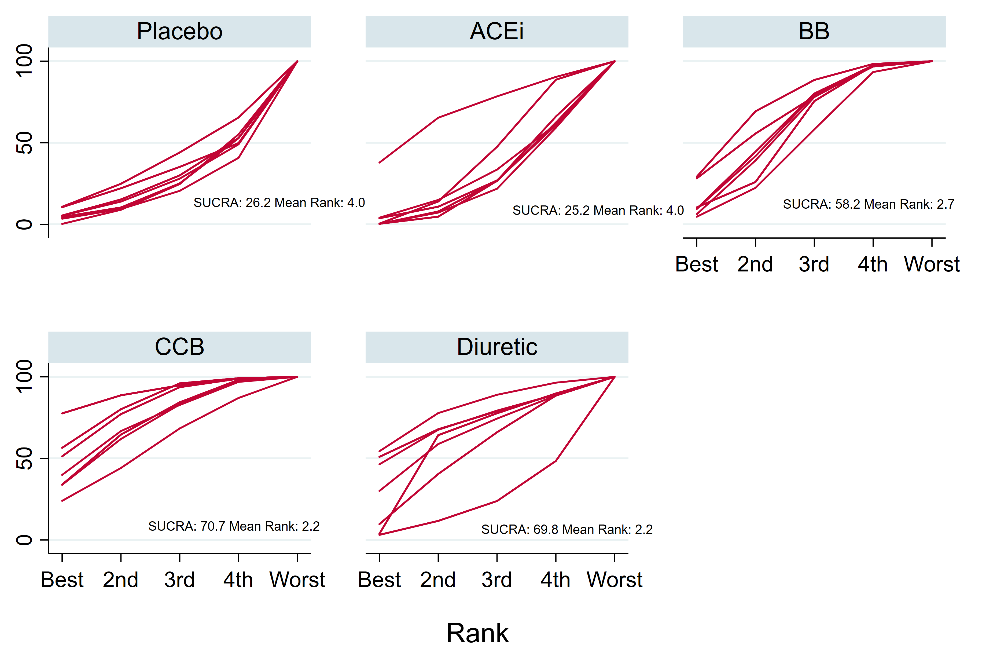


Test of consistency

chi^2^ =2.55

p = 0.86

**Additional File 3: Figure S4**

Funnel plot for global meta-analysis of 31 studies, grouped by blood pressure agent class. Effect size refers to reported change in mean systolic blood pressure (mmHg). Pseudo 95% confidence intervals plotted. Egger’s test for publication bias across all studies: slope = 0.524 (-2.32 to 3.36), intercept or bias = -0.200, p=0.713.


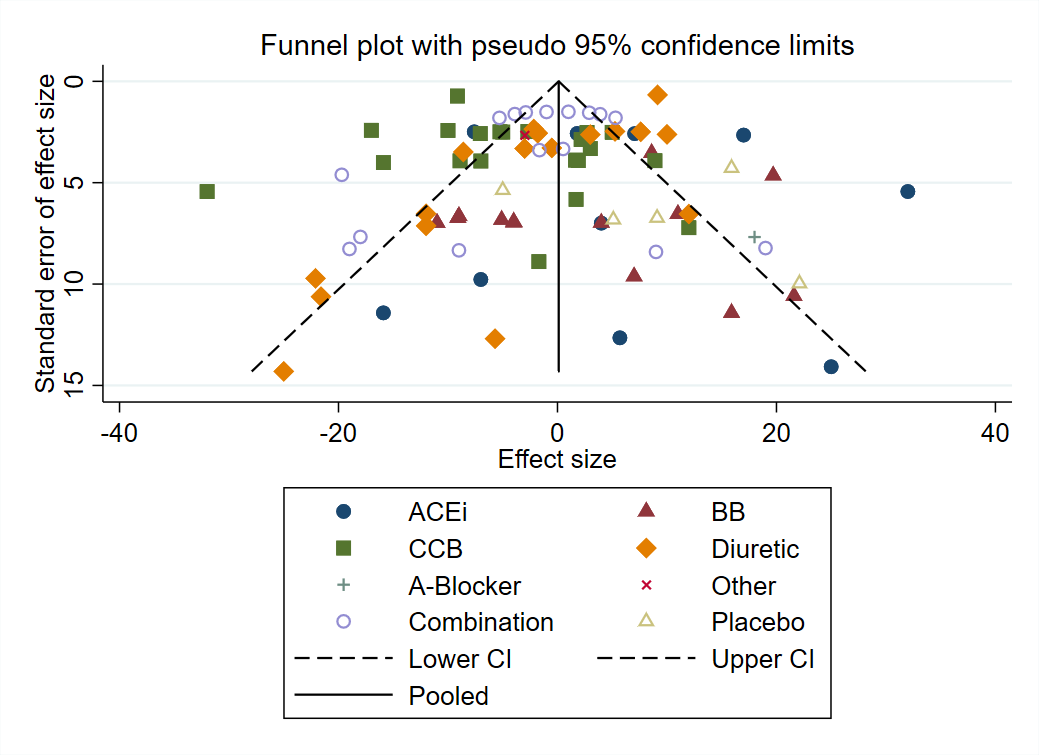

Supplement: Supplementary file 3 — Additional file 3: Supplementary Figures of additional results not displayed in the infographics of the main text. Figure S1. Forest Plots showing results of network meta-analysis of monotherapy on systolic and diastolic blood pressure. Figure S2. Trends in blood pressure lowering efficacy of treatment with (a) age, (b) gender and (c) number of participants and (d) year of publication. Figure S3. Rankograms for Network Meta-analysis with exclusion of imputed missing values (a) SBP (b) DBP and exclusion of studies rates as high risk of bias (c) SBP and (d) DBP. Figure S4. Funnel plot for global meta-analysis of 31 studies, grouped by blood pressure agent class. [file 12916_2020_1530_MOESM3_ESM.docx]
